# Comprehensive Survey of PCV2 and PCV3 in Domestic Pigs and Wild Boars Across Portugal: Prevalence, Geographical Distribution and Genetic Diversity

**DOI:** 10.3390/pathogens14070675

**Published:** 2025-07-09

**Authors:** Bernardo Almeida, Margarida D. Duarte, Ana Duarte, Teresa Fagulha, Fernanda Ramos, Tiago Luís, Inês Caetano, Sílvia C. Barros, Fábio Abade dos Santos, Ana Margarida Henriques

**Affiliations:** 1 National Institute of Agrarian and Veterinarian Research, Quinta Do Marquês, Av. da República, 2780-157 Oeiras, Portugal; bsa.almeida2002@gmail.com (B.A.); margarida.duarte@iniav.pt (M.D.D.); ana.duarte@iniav.pt (A.D.); teresa.fagulha@iniav.pt (T.F.); fernanda.ramos@iniav.pt (F.R.); tiago.luis@iniav.pt (T.L.); ines.caetano@iniav.pt (I.C.); silvia.santosbarros@iniav.pt (S.C.B.); fabio.abade@iniav.pt (F.A.d.S.); 2 Faculdade de Medicina Veterinaria, Centre for Interdisciplinary Research in Animal Health (CIISA), Universidade de Lisboa, Avenida da Universidade Tecnica, 1300-477 Lisboa, Portugal; 3 Associate Laboratory for Animal and Veterinary Sciences (AL4AnimalS), Avenida da Universidade de Lisboa, 1300-477 Lisboa, Portugal; 4 Faculdade de Ciências e Tecnologia, Departamento de Ciências da Vida, Universidade Nova de Lisboa, Campus da Caparica, 2829-516 Caparica, Portugal; 5 CECAV—Centro de Ciência Animal e Veterinaria, Faculdade de Medicina, Veterinaria de Lisboa, Universidade Lusofona, Centro Universitario de Lisboa, 1749-024 Lisboa, Portugal

**Keywords:** *Porcine Circovirus type 2* (PCV2), *Porcine Circovirus type 3* (PCV3), viral prevalence, coinfection, epidemiology, geographical distribution

## Abstract

Porcine circoviruses are significant pathogens that affect swine populations worldwide, with implications for animal health and productivity. While PCV2 is well-documented, particularly due to widespread vaccination programs, PCV3 is less understood, and its epidemiological impact is still under investigation. This study screened for PCV2 and PCV3 in pigs and wild boars across Portugal to assess their prevalence. Also, nucleotide sequence determination was performed to evaluate the genetic diversity of these viruses. Stool samples from 160 pigs belonging to different groups (quarantine, nursery, fattening and adult pigs), as well as organ samples from 120 hunted wild boars, were analyzed. Samples were collected from twelve of the eighteen mainland Portuguese districts with positive cases being detected in nine of them. Pigs had a lower prevalence of PCV2 (1.9%) than PCV3 (11.2%), but the opposite was true in wild boars (76.7% for PCV2 and 55.0% for PCV3). The lower PCV2 prevalence in pigs can be attributed to the PCV2 vaccination program implemented. Additionally, these viruses were significantly more prevalent in wild boars (90.8% were infected with at least one of the viruses) than in domestic pigs (only 12.5%). This significant difference highlights the impact of the controlled environment in pig farms on disease prevention in contrast to the higher exposure risks faced by wild boars in their natural habitat. Compared to a previous study from 2023, we observed a slight decrease in the percentage of positive cases for both PCV2 and PCV3. Phylogenetic analysis of sequences obtained by Sanger sequencing allowed us to conclude that the samples from domestic pigs belong to the PCV2a and PCV3c clades, in contrast to the PCV2-positive cases detected in domestic pigs in 2023 that were classified in the PCV2d genotype. Conversely, samples from wild boars belong to the PCV2d and PCV3a clades. These results reveal genotype differences between wild and domestic pigs and shifts from 2023 to 2024. Our findings provide some information about the circulation of these viruses and emphasize the importance of vaccination and continued monitoring for a deeper understanding of their epidemiology to mitigate potential risks to swine health and production.

## 1. Introduction

Porcine circoviruses are among the most significant viral agents affecting global swine health. They have implications for animal welfare, productivity and economic sustainability. The *Circoviridae* family contains small, non-enveloped, circular single-strand DNA viruses that can infect swine, including domestic pigs and wild boars. PCVs primarily target lymphoid tissues, weakening the immune system and facilitating secondary infections [1,2]. PCVs are primarily transmitted via the fecal–oral route but can also spread through direct contact, vertical transmission and exposure to bodily fluids from infected animals [3].

*Porcine circovirus type 2* (PCV2) is the most studied virus in this family due to its association with porcine-circovirus-associated disease (PCVAD). PCVAD is a complex of syndromes that cause severe illness and reproductive disorders in swine [4]. In 2016, a new member of the *Circoviridae* family emerged: *porcine circovirus type 3* (PCV3). It was first reported in the United States and later in several other locations, including Asia and European countries such as Italy [5], Spain [6], Poland [7] and Portugal [8]. PCV3 diverges from PCV2, sharing less than 50% nucleotide identity in the capsid gene and 26–36% amino acid identity in the replicase gene [9]. The PCV3 genome contains two main open reading frames (ORFs): ORF1 encoding the replicase (Rep) and ORF2 encoding the capsid protein (Cap) and a putative ORF3, similar to other circoviruses like PCV2 [9].

While the pathogenic potential of PCV3 remains under investigation, it has been detected in both symptomatic and asymptomatic animals, suggesting that it may play a role in subclinical infections or act as a cofactor in disease development [10]. PCV2 has been effectively controlled in many commercial pig populations through widespread vaccination, leading to a significant reduction in clinical disease and viral prevalence [11,12]. Inactivated vaccines are available from a number of companies against PCV2. The dose to be applied and the age of the animal vary from company to company. Available vaccines target PCV2a and PCV2b genotypes and have cross-protective efficacy against other variants such as PCV2d [13,14,15]. However, there is currently no available vaccine for PCV3, and its epidemiological dynamics are still understudied. This knowledge gap raises concerns about PCV3 circulation, particularly in regions where it coexists with PCV2.

The role of wild boars (*Sus scrofa scrofa*) as reservoirs for swine pathogens is receiving increased attention. Their growing populations and potential to interact with domestic herds, either directly or through shared environments, pose challenges to biosecurity and disease control [16]. Indeed, a recent study found PCV2 and PCV3 in wild boar reproductive systems in 14.3% of samples.

Recent studies have shown that PCV2 remains in circulation in the swine population despite control measures implemented in Portugal through vaccination programs since 2007 [8,12].

The main goal of this study is to evaluate the prevalence of PCV2 and PCV3 in domestic pigs and wild boars in mainland Portugal s (s. By examining both populations and including multiple tissue types and production stages, this research contributes to a better understanding of virus circulation, the impact of vaccination and potential interactions between PCV2 and PCV3.

## 2. Materials and Methods

### 2.1. Sample Collection and Nucleic Acid Extraction

From August to December 2024, feces from 160 domestic pigs were collected from domestic pig farms in seven districts of mainland Portugal (Santarém, Lisbon, Leiria, Setúbal, Aveiro, Faro and Coimbra). These samples were categorized into four different groups, according to the production stage of the animals, quarantine (n = 5), nursery (n = 24), fattening (n = 28) and adult pigs (n = 103). Additionally, 120 organ samples from various tissues (liver, spleen, bone marrow, lungs, retropharyngeal lymph nodes, submandibular lymph nodes and diaphragm) were collected from wild boars in seven districts (Santarém, Évora, Beja, Aveiro, Guarda, Castelo Branco and Portalegre) from October 2023 to February 2025. The selection of different districts for sampling domestic pigs and wild boars, with only two overlapping, enabled full coverage of the southern and central regions of mainland Portugal. Both domestic pig and wild boar samples were received by the laboratory within the scope of other studies, namely hepatitis E virus or tuberculosis screening, respectively.

Sample preparation involved diluting a small amount of feces to 20% (*w*/*v*) in phosphate-buffered saline (PBS). The suspension was vortexed to ensure thorough homogenization, then centrifuged at 2000× *g* for 10 min to remove debris. The organ samples were homogenized at 20% (*w*/*v*) in PBS in a Precellys tissue homogenizer (Bertin Technologies, Montigny-le-Bretonneux, France) with zirconium spheres and clarified at 2000× *g* for 5 min. Nucleic acids were extracted using the IndiMag Pathogen Kit (Indical, Leipzig, Germany) on the KingFisher Flex nucleic acid extraction system (ThermoFisher Scientific, Waltham, MA, USA) according to the manufacturer’s protocol.

### 2.2. qPCR for PCV2 and PCV3 Detection

For both PCV2 and PCV3, the reaction mix included 1 µM of forward primer, 1 µM of reverse primer, 0.2 µM of probe and 12.5 µL of buffer (SPEEDY NZYTaq 2x Colourless Master Mix from NZYTech, Lisbon, Portugal). For PCV2, 5 µL of DNA was used, while for PCV3, only 2.5 µL of sample was used. The total volume was adjusted to 25 µL with nuclease-free water. Primer sequences are listed in Table 1. A positive field sample was included in each reaction as a positive control.

The qPCR amplification program performed to detect the PCV2 viral *rep* gene consisted of an initial denaturation at 95 °C for 2 min, followed by 45 cycles of denaturation at 95 °C for 2 s, annealing at 52 °C for 5 s and extension at 72 °C for 5 s. For the PCV3 qPCR, targeting the *cap* gene, the amplification program included an initial denaturation at 95 °C for 2 min, followed by 40 cycles of denaturation at 95 °C for 2 s and an annealing/extension step at 60 °C for 5 s.

The viral load was inferred from the cycle threshold (Ct) values, with lower Ct values indicating higher amounts of viral DNA present in the sample, as fewer amplification cycles are needed to reach the detection threshold.

### 2.3. Conventional PCR Amplification of Positive qPCR Samples

Four PCV2-positive samples (three from pigs and one from a wild boar) and one PCV3-positive sample from a wild boar were selected for Sanger sequencing based on their collection sites in order to infer genetic differences between different districts in mainland Portugal.

PCV2 reaction mix included 1 µM primer S4, 1 µM primer AS4 (Table 1), 12.5 µL of NZYTaq II 2x Green Master Mix (NZYTech, Lisbon, Portugal), 5 µL DNA and water to a total volume of 25 µL. The amplification program started with an initial denaturation step at 95 °C for 2 min, followed by 40 cycles of denaturation at 95 °C for 30 s, annealing at 58 °C for 30 s, extension at 72 °C for 90 s and a final extension step at 72 °C for 5 min. The amplified fragment has an expected size of 494 bp.

PCV3 reaction mix included 1 µM primer PCV3_seq2_FW, 1 µM primer PCV3_seq2_RV (Table 1), 12.5 µL of NZYTaq II 2x Green Master Mix (NZYTech, Lisbon, Portugal), 2.5 µL of DNA and water to a total volume of 25 µL. The amplification program started with an initial denaturation step at 95 °C for 2 min, followed by 45 cycles of denaturation at 95 °C for 30 s, annealing at 56 °C for 30 s and extension at 72 °C for 1 min and a final extension step at 72 °C for 5 min. The amplified fragment has an expected size of 825 bp.

The amplification products were observed in 1% agarose gel electrophoresis with GreenSafe (NZYTech, Lisbon, Portugal) in a GelDoc Go Imaging System (BioRad, Hercules, CA, USA) and the specific products were excised and purified using the NZYGelpure kit (NZYTech, Lisbon, Portugal). Primer sequences are listed in Table 1.

### 2.4. Sanger Sequencing

Sanger sequencing was carried out using the BigDye Terminator Cycle Sequencing Kit (Applied Biosystems, Foster City, CA, USA) following the manufacturer’s protocol. The forward and reverse primers from the amplification step were used. Each reaction (10 µL total volume) included 1 µL of sequencing buffer, 2 µL of sequencing mix, 2.5 µM primer and an appropriate volume of DNA, adjusted based on fragment concentration. The sequencing protocol consisted of an initial denaturation at 96 °C for 1 min, followed by 25 cycles of denaturation at 96 °C for 10 s, annealing at 58 °C for PCV2 or 56 °C for PCV3 for 5 s and extension at 60 °C for 1 min. The products were purified using sodium acetate (3M, pH 5.2), 125 mM EDTA and 100% ethanol. After drying, samples were resuspended in formamide and sequenced using a 3130 Genetic Analyzer (Applied Biosystems, Waltham, MA, USA). Sequence reads were assembled with SeqScape v2.5 software (Applied Biosystems).

Nucleotide sequences obtained were submitted to GenBank using BankIT and received accession numbers PV753264–PV753268.

### 2.5. Phylogenetic Analysis

To identify the genotypes of PCV2 and PCV3 strains circulating in domestic pigs and wild boars in Portugal, the sequences obtained were aligned with representative GenBank sequences originated from different hosts, regions and years. Multiple sequence alignments were performed using the AliView program (version 1.28). A phylogenetic tree of PCV2 was made by IQ-TREE (http://iqtree.cibiv.univie.ac.at/, accessed on 6 July 2025) with the best-fit substitution model selected automatically by the software, namely the TIM2 + F + I + G4 (Transitional model 2) model. For PCV3, the best-fit substitution model was selected in MEGA X (v10.0.5) based on model selection criteria. A phylogenetic tree was also constructed in MEGA X, using the maximum likelihood method and K2 + G model. Initial trees for heuristic searches were generated using the neighbor-joining algorithm with distances estimated via the maximum composite likelihood method. Site-specific rate heterogeneity was modeled using a discrete gamma distribution with invariant sites allowed. The bootstrap method was used with 1000 bootstrap replications.

## 3. Results

A total of 160 feces samples were collected from domestic pigs. In contrast, the 120 samples from wild boars were collected from various tissues, including the liver, spleen, bone marrow, lungs, retropharyngeal lymph nodes, submandibular lymph nodes and diaphragm. The presence of PCV2 and PCV3 viruses was detected in all types of analyzed tissues (Table 2).

Of the 280 swine tested, 33.93% (n = 95) were infected with PCV2 and 30.00% (n = 84 were infected) with PCV3. Coinfection was detected in 1 domestic pig and in 52 wild boars. While 87.50% (n = 140) of domestic pigs tested negative for both viruses, 3 pigs positive for PCV2, 18 for PCV3 and 1 coinfection case were detected. In contrast, only 11.67% (n = 14) of wild boars tested negative for PCV2 and PCV3 (92 tested positive for PCV2, 66 for PCV3 and 52 for both).

The positivity rate in domestic pigs varied by production stage, with PCV2 present only in early stages of production (nursery and fattening), while PCV3 was found in all groups (Table 2).

The chi-square test was applied to the results obtained. Since the chi-square value exceeds the critical value (21.92 > 5.991), the null hypothesis (there is no association between the host and the type of virus) was rejected, indicating a significant association between the type of animal and the type of PCV infection. A chi-square test was also conducted to compare the pig production stage and the detection of PCV2, PCV3 or coinfection, leading to the conclusion that there is no statistically significant association between the pig production stage and the infection type (PCV2, PCV3, coinfection) at the 5% significance level (χ^2^ = 6.32 < 12.592).

Compared to a 2023 study [8], which focused solely on domestic pigs, both PCV2 and PCV3 infection rates appear to have declined: PCV2 decreased from 3.2% to 1.9% and PCV3 decreased from 19.4% to 11.2%. Concerning wild boar, samples from 2023 to 2025 were included in this study. The results were similar, showing a decrease in infection rates for both viruses: PCV2 declined from 93.3% in 2023 to 71.2% in 2024, and PCV3 showed a small reduction from 66.7% to 51.7. The coinfection level declined from 63.3% to 37.1%. These results are summarized in Table 2.

**Figure 1:**
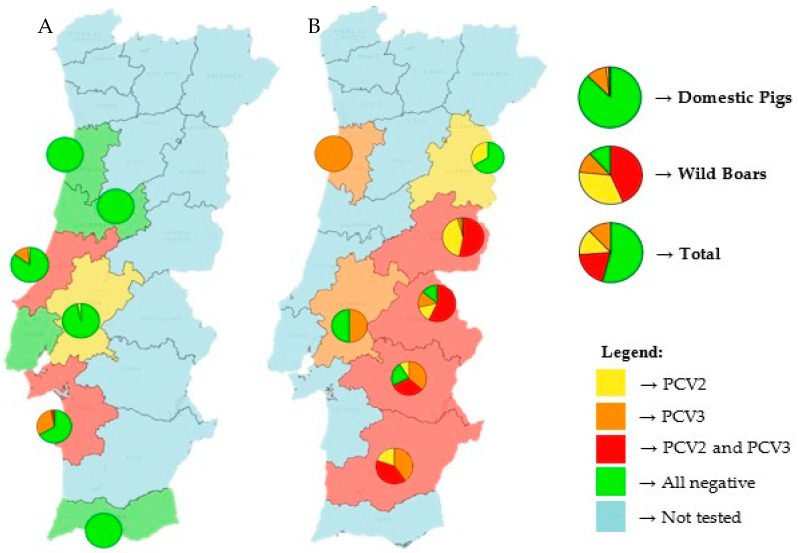
Geographic distribution and prevalence of PCV2 and PCV3 in domestic pigs (**A**) and wild boars (**B**) in mainland Portugal. Districts are color-coded according to virus presence: yellow for PCV2-positive, orange for PCV3-positive, red for PCV2 and PCV3 coinfection, green for negative results and blue for untested districts. On top of each tested district, a pie chart illustrates the proportions of PCV2-positive (yellow), PCV3-positive (orange), coinfection (red) and negative (green) samples. On the right side of the map, three larger pie charts display the same viral distribution by host category: domestic pigs (top), wild boars (middle) and the combined total of both populations (bottom).

Figure 1 shows the distribution of both viruses in Portugal. Nine of the twelve tested mainland Portuguese districts had at least one virus present, with only three districts (Aveiro, Lisbon and Faro) showing no detection of either virus. Additionally, a higher prevalence of both viruses can be seen in eastern districts, likely because most of the tested wild boars came from these regions. The districts of Castelo Branco, Portalegre, Évora and Beja displayed the highest prevalence of coinfection, exceeding 50% in some cases.

In the districts where only domestic pigs were tested, it is important to note that one-third of the animals in the districts of Setúbal and Leiria were infected, primarily with PCV3.

Among domestic pigs, PCV3 was the most prevalent virus (11.2%), followed by low rates of PCV2 (1.9%) and coinfection (0.6%). In contrast, wild boars had markedly higher detection rates for all categories: 76.7% for PCV2, 55.0% for PCV3 and 43.3% for coinfections.

A comparative analysis of the Ct values obtained for PCV2 and PCV3 in wild boars and domestic pigs was conducted using box-and-whisker plots (Supplementary Figure S1).

Although comparisons of Ct values obtained with different qPCR systems are not totally reliable, the Ct values obtained with positive PCV2 samples from wild boars were significantly lower than those obtained with PCV3-positive wild boar samples, suggesting a higher viral charge of PCV2 in this host. In domestic pigs, PCV2 viral charges are also higher than those of PCV3, however, due to the small number of PCV2-positive domestic pigs, this difference is not statistically significant.

The comparison of viral charges between wild boars and domestic pigs is not reliable, since different matrices were used (organs for wild boars and feces for domestic pigs). However, although no direct studies have been published on PCV2 and PCV3, research on related viruses that infect domestic pigs and wild boars, such as PCV4 and HEV, found in Refs. [20,21], has shown that Ct values obtained via qPCR from feces and organs, such as the liver, kidneys, diaphragm, spleen and lungs, are comparable within the same animal. These results suggest that viral load assessments are consistent across these sample types, supporting the assumption that PCV2 and PCV3 behave similarly in terms of viral load distribution.

For PCV2, however, robust comparisons are limited due to the small number of positive cases detected in domestic pigs (n = 3). However, it is noteworthy that the average Ct values were similar between wild boars and domestic pigs (25.84 and 25.00, respectively).

In contrast, the average PCV3 Ct value was higher in domestic pigs (33.75) than in wild boars (31.15), as was the median (34.75 and 29.81, respectively). This suggests a lower viral load in the domestic pig population. However, this difference is not statistically significant (*p* > 0.05), as determined by a one-way ANOVA followed by a Tukey’s honestly significant difference (HSD) test using IBM SPSS software (https://www.ibm.com/products/spss, accessed on 6 July 2025).

**Figure 2:**
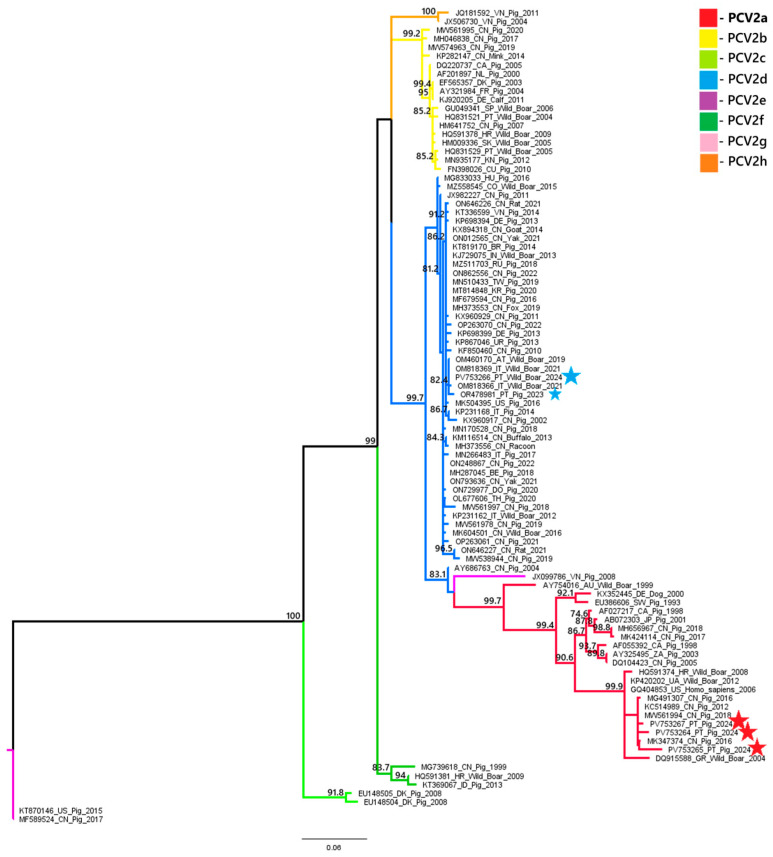
Evolutionary analysis of PCV2 inferred in IQ-TREE by using the Maximum Likelihood method and TIM2 + F + I + G4 (Transitional model 2) model [22]. The tree with the highest log likelihood (−2907.499) is shown. The phylogenetic analysis was performed using a 494 nt fragment of the capsid gene. Sequences marked with a big star represent samples obtained in the present study. The sequence marked with a small blue star originated from a sample of a previous study.

**Figure 3:**
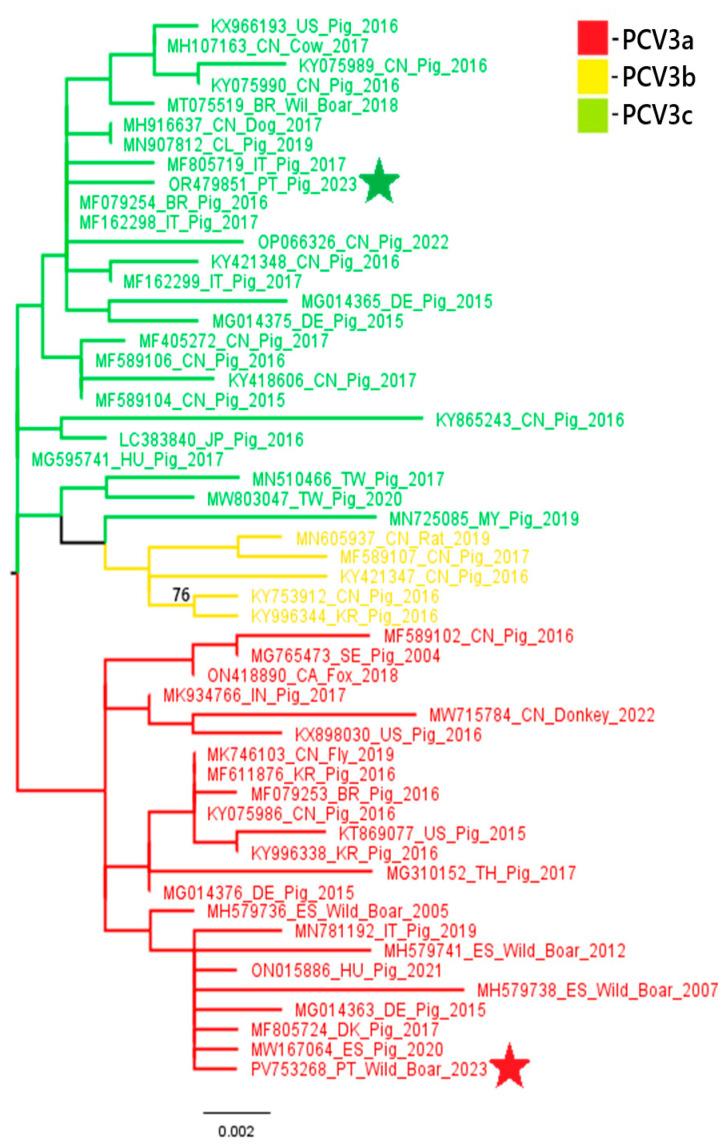
The evolutionary history was inferred by using the Maximum Likelihood method and Kimura 2-parameter model [23]. The tree with the highest log likelihood (−1976.04) is shown. The percentage of trees in which the associated taxa clustered together is shown next to the branches. Initial tree(s) for the heuristic search were obtained automatically by applying Neighbor-Joining and BioNJ algorithms to a matrix of pairwise distances estimated using the Maximum Composite Likelihood (MCL) approach and then selecting the topology with superior log likelihood value. A discrete Gamma distribution was used to model evolutionary rate differences among sites (5 categories (+G, parameter = 0.1238)). The tree is drawn to scale, with branch lengths measured in the number of substitutions per site. This analysis involved 54 nucleotide sequences. Codon positions included were 1st + 2nd + 3rd + Non-coding. After removing the primer sequences, there were a total of 787 positions in the final dataset. Evolutionary analyses were conducted in MEGA X [24]. Sequences marked with a red and a green star represent samples obtained in the present study and in the previous study, respectively.

A phylogenetic analysis of PCV2 and PCV3 nucleotide sequences revealed distinct genotypic distributions between domestic pigs and wild boars. All three PCV2 sequences obtained from domestic pigs (samples 18743, 18918 and 22355) belonged to the PCV2a genotype. In contrast, the PCV2 sequence from a wild boar (sample 20063) belonged to the PCV2d genotype. These results suggest that there is diversity in genotypes between the two populations. Similarly, the PCV3 sequence from the domestic pig (sample L2.6) belonged to PCV3c, while the wild boar sequence (sample 03762.1) was classified as PCV3a. These results indicate the circulation of different PCV3 genotypes across hosts. These findings are illustrated in Figure 2 and Figure 3.

## 4. Discussion

This study provides new insights on the epidemiology and dynamics of PCV2 and PCV3 in wild boars and domestic pigs in different regions of mainland Portugal. A key observation in this study is the low PCV2 infection rate among domestic pig populations suggesting the effectiveness of PCV2 vaccination programs. However, other reasons may contribute to this low prevalence, such as a low LOD or mismatches between the primers/probe and the viral sequence. It is important to notice that prevalence can also vary by the type of PCR used, extraction efficiency and the type of matrix used. Within domestic pigs, viral distribution varied according to production stage, with PCV2 being only detected in nursery and fattening pigs (pigs less than six months old), which aligns with previous studies showing early-life susceptibility to PCV2 infection, possibly due to waning maternal antibodies [25]. In domestic pigs, the PCV3 infection rate was significantly higher than that of PCV2 infection (1.9% for PCV2 and 11.2% for PCV3) and PCV3 was found across all production stages, suggesting a broader distribution or a longer persistence within domestic herds. This finding contrasts with the situation in wild boar populations, where the PCV2 infection rate reaches 76.7%, probably due to the absence of vaccination in these wild populations. The prevalence of both PCV2 and PCV3 was higher in wild boars than in domestic pigs. While only 12.5% of domestic pigs tested positive for at least one virus, 88.3% of wild boars were infected with PCV2, PCV3 or both. The prevalences obtained were much higher than those found in wild boars by others [26]. These differences probably reflect the lack of vaccination in wild boar populations, as well as potential differences in environmental exposure, showing how farm biosecurity policies effectively limit pathogen spread.

Concerning PCV3, a similar infection rate was expected in both domestic pigs and wild boars as they are not vaccinated against this virus in Portugal. However, the observed discrepancy raises questions about a putative interplay between PCV2 vaccination and PCV3 infection in domestic pigs. Although no direct evidence was observed in this study, the lower prevalence of PCV3 in domestic pigs compared to wild boars suggests the possibility that PCV2 vaccination might indirectly influence PCV3 infection dynamics. However, this remains speculative, as serological testing or experimental validation was not conducted. Nonetheless, while cross-protection remains a possibility, other factors may also contribute to this discrepancy, such as differences in biosecurity measures [27], levels of animal confinement [28] and frequency of contact with environmental sources of infection [27,29]. Domestic pigs, which are raised in controlled farm settings, are less likely to encounter diverse viral sources compared to wild boars, which may have extensive contact with contaminated habitats or other wildlife.

Coinfection, especially in wild boars, remains an important concern (43.3%). The high rate of coinfection found in wild boars raises questions about their role as PCV2 and PCV3 reservoirs and sources for ongoing transmission to domestic pigs. Notably, differences in coinfection rates and viral loads were observed across host species and production stages, which may be influenced not only by host- or age-related factors but also by the different sample types analyzed (organs in wild boars and feces in domestic pigs). Although previous research on related viruses [20,21] suggests comparable viral loads across feces and organ samples within the same animal, this difference in sampling could still impact viral load assessments and should be considered when interpreting the results.

A comparison with a previous study conducted in 2023 revealed a decrease in the infection rates of both PCV2 and PCV3 in the pig population with PCV2 positivity dropping from 3.2% to 1.9% and PCV3 from 19.4% to 11.2%. In the wild boar population, the prevalence of these viruses behaved similarly when comparing 2023 with 2024 samples, showing a reduction in the PCV2 (from 93.3% to 71.2%) and PCV3 (from 66.7% to 51.7%) prevalence with a notable decline in coinfection rates (from 63.3% to 37.1%). In both studies, the samples collected from pigs were feces samples, and the tested animals represented different production stages, which supports this temporal comparison. These trends may reflect real epidemiological changes as a result of improved biosecurity measures in farms or temporal variation.

Geographically, the presence of the two viruses was detected in 9 out of 12 sampled districts of mainland Portugal. The eastern regions, particularly Castelo Branco, Portalegre, Évora and Beja, exhibited the highest coinfection rates, surpassing 50% in certain areas. This is likely due to the greater number of wild boars sampled from these districts. Interestingly, notable levels of coinfection were observed even in some districts where only domestic pigs were tested, such as Setúbal and Leiria. These results highlight the importance of regional monitoring and indicate possible environmental or management-related risk factors.

Viral load differences were evaluated based on the analysis of the Ct values obtained. Although sample types differed (feces in pigs vs. tissues in wild boars), existing literature on similar viruses [20,21] supports the comparability of qPCR Ct values across these matrices. PCV3 Ct values were significantly higher in domestic pigs than in wild boars, suggesting a lower viral load in the domestic population. For PCV2, robust comparisons were limited due to the small number of positive domestic pig samples (n = 3), but average Ct values were similar between the pig groups. These observations reinforce the value of viral load analysis for understanding infection dynamics, while also emphasizing the need for more extensive sampling in domestic pigs.

Although vaccination does not prevent infection, PCV2 vaccination may influence the immune response in pigs, potentially reducing PCV3 replication and viral load, providing a quicker disease recovery and virus elimination. This could potentially explain the lower prevalence of PCV3 in domestic pigs compared to wild boars, alongside the effect of farm management and sanitary practices. This would also explain the higher Ct values observed in domestic pigs compared to wild boars. Nonetheless, this hypothesis requires experimental confirmation in future studies to fully understand the interactions between PCV2 and PCV3 in terms of viral dynamics and immune responses. Further research is needed to better understand the immune dynamics and viral interactions during PCV2 and PCV3 infections, as supported by the trend differences in Ct values between the two populations and this apparent interference.

Phylogenetic analysis of the PCV2 and PCV3 nucleotide sequences obtained in this study suggests some variation in genotype distributions between different host species. All three PCV2 sequences obtained from domestic pigs were classified as PCV2a, while the wild boar sequence was identified as PCV2d. This contrasts with the previous data from 2023 [8], where the PCV2 genotype circulating in domestic pigs was PCV2d. Although limited by the small number of sequenced samples, the absence of PCV2d in the current pig samples may showcase a shift in genotype prevalence or sampling variation due to the limited number of sequenced samples. Concerning PCV3, our findings are more consistent with previous data: the domestic pig sample clustered within the PCV3c genotype, aligning with the dominant strain reported in pigs in 2023 [8], while wild boar positive samples belonged to PCV3a, a genotype previously identified in wildlife in other European countries, namely in Italy, Sardinia and in Germany [30,31]. Noting, once more, the limited number of sequenced samples, these findings suggest that certain PCV3 genotypes may be more common in specific hosts. However, further studies with larger sample sizes and broader genomic coverage are necessary to confirm any host-associated genotype patterns. This host-specific circulation of genotypes, with PCV3c potentially adapting better to domestic pigs (subspecies *Sus scrofa domesticus*) and PCV3a being more prevalent in wild boar populations (species *Sus scrofa scrofa*), should be interpreted with caution and validated by future studies. These findings, although limited by the small number of samples sequenced, raise the possibility of genotype segregation between domestic and wild populations and emphasize the importance of continuous molecular surveillance. Future studies with larger PCV sequence datasets from domestic and wild Suidae will help trace the emergence and spread of the different genotypes, explore the role of wild boars in virus transmission and evaluate how factors like vaccination and environmental persistence may influence viral evolution. The detection of distinct genotypes circulating independently in wild boars may reflect host-specific viral evolution or different transmission networks. This finding underscores the importance of ongoing surveillance of PCV2 and PCV3 in domestic and wild pig populations, particularly in areas with higher coinfection rates, to identify the circulating genotypes, which will provide additional context for the current laboratory findings.

Overall, this study highlights the continued circulation of PCV2 and PCV3 in both domestic pigs and wild boars in Portugal, with wild boars showing significantly higher infection and coinfection rates than domestic pigs. These findings emphasize the importance of integrating wildlife surveillance into national swine health programs and provide a foundation for future studies investigating transmission dynamics, control strategies and the potential impact of these viruses on animal health and production.

## 5. Conclusions

In summary, this study provides some information on the viral dynamics of PCV2 and PCV3 in domestic pigs and wild boars in mainland Portugal, suggesting a possible impact of PCV2 on PCV3 infections due to vaccination programs. The results obtained suggest not only the effectiveness of the PCV2 vaccination program in domestic pigs but also that vaccination of domestic pigs against PCV2 could reduce PCV3 viral load during infection, potentially by limiting virus replication. PCV2a and PCV3c genotypes were identified in domestic pig samples, whereas PCV2d and PCV3a genotypes were identified in wild boar samples. In addition to industrial domestic pigs and wild boars, investigations should target traditional free-range Iberian pigs, as they represent a critical epidemiological interface between the two groups. These animals often share habitats with wild boars while maintaining indirect links to intensive farming systems through human management, making them a potential epidemiological bridge for the transmission of pathogens. This approach is essential to confirm the hypothesis that there is an apparent segregation of genotypes among domestic and wild pigs. Investigations should also focus on exploring potential cross-virus interactions and the implications of PCV2 and PCV3 genotypes on pig health, disease management and meat production efficiency.

These findings highlight the importance of monitoring both domestic and wild populations to better understand the epidemiology and potential transmission pathways of porcine circoviruses.

## Figures and Tables

**Table 1:** Primer and probe sequences used for the detection of PCV2 and PCV3 by qPCR and conventional PCR. For each reaction, the corresponding forward (F), reverse (R) and probe (when applicable) sequences are listed in 5′ to 3′ orientation. Fluorescent labeling with FAM was used for qPCR probes.

Reaction	Name	Sequence	Reference	Target Gene
qPCR PCV2	PCV2-PT-rep6(F)	5′-CAGCAAGAAGAATGGAAG-3′	[17]	Replicase
	PCV2-PT-rep149(R)	5′-TTACCCTCCTCGCCAAC-3′		
	PCV2-PT probe(R)	5′-[FAM]TCCCGTATTTTCTTGCGCTCGTCTTC-3’		
qPCR PCV3	PCV3_real_FW	5′-AGTGCTCCCCATTGAACG-3′	[18]	Capsid
	PCV3_real_RV	5′-ACACAGCCGTTACTTCAC-3′		
	PCV3_real_probe	5′-[FAM]ACCCCATGGCTCAACACATATGACC-3′		
PCR PCV2	S4	5′-CACGGATATTGTAGTCCTGGT-3′	[19]	Capsid
	AS4	5′-CCGCACCTTCGGATATACTGTC-3′		
PCR PCV3	PCV3_seq2_FW	5′-GTCGTCTTGGAGCCAAGTG-3′	[18]	Capsid/Replicase
	PCV3_seq2_RV	5′-CGACCAAATCCGGGTAAGC-3′		

**Table 2:** Distribution of PCV2, PCV3 and coinfection cases among different pig production stages and wild boars. The number of tested animals, age range and infection rates (in absolute numbers and percentages) are indicated for each group. Percentages represent the proportion of infected animals within each group.

Group	Epidemiological Unit	Year of Sampling	Age	Number of Tested Animals	PCV2 Infection	PCV3 Infection	PCV2 and PCV3 Coinfection
Domestic pig	Quarantine	2024	Any	5	0	1 (20.0%)	0
	Nursery		3 to 10 weeks	24	1 (4.2%)	3 (12.5%)	1 (4.2%)
	Fattening		10 weeks to 6 months	28	2 (7.1%)	3 (10.7%)	0
	Adult pigs		More than 7 months	103	0	11 (10.7%)	0
	**Total pigs**		-	160	3 (1.9%)	18 (11.2%)	1 (0.6%)
Wild boar	N/A	2023	Unknown	30	28 (93.3%)	20 (66.7%)	19 (63.3%)
		2024	Unknown	89	64 (71.2%)	46 (51.7%)	33 (37.1%)
		2025	Unknown	1	0	0	0
	**Total wild boars**		-	120	92 (76.7%)	66 (55.0%)	52 (43.3%)

## Data Availability

The data supporting the results of this study can be obtained by contacting the corresponding author, however, the right to privacy of the property owners will be respected.

## Supplementary Materials

The following supporting information can be downloaded at: https://www.mdpi.com/article/10.3390/pathogens14070675/s1, Figure S1: Box-and-whisker plots showing qPCR Ct values of positive samples from wild boar and domestic pigs for PCV2 and PCV3.

## Abbreviations

The following abbreviations are used in this manuscript:

°C	Degrees Celsius
µL	Microliter
µM	Micromolar
bp	Base pairs
Ct	Cycle threshold
F	Forward
FAM	6-carboxyfluorescein
HEV	Hepatitis E virus
HSD LOD	Honestly significant difference Limit of detection
PBS	Phosphate-buffered saline
PCR	Polymerase chain reaction
PCV2	*Porcine circovirus type 2*
PCV3	*Porcine circovirus type 3*
PCV4	*Porcine circovirus type 4*
PCVAD	Porcine-circovirus-associated disease
qPCR	Quantitative polymerase chain reaction
R	Reverse
*w*/*v*	Weight per volume
×g	Relative centrifugal force (RCF)
