# Supplementary material for: Comprehensive Survey of PCV2 and PCV3 in Domestic Pigs and Wild Boars Across Portugal: Prevalence, Geographical Distribution and Genetic Diversity
Source: Pathogens. 2025 Jul 9;14(7):675. doi: 10.3390/pathogens14070675 (PMC12300804; doi:10.3390/pathogens14070675)
Supplement: Supplementary file 1 [file pathogens-14-00675-s001.zip › pathogens-3703184-supplementary.pptx]

## Slide 1
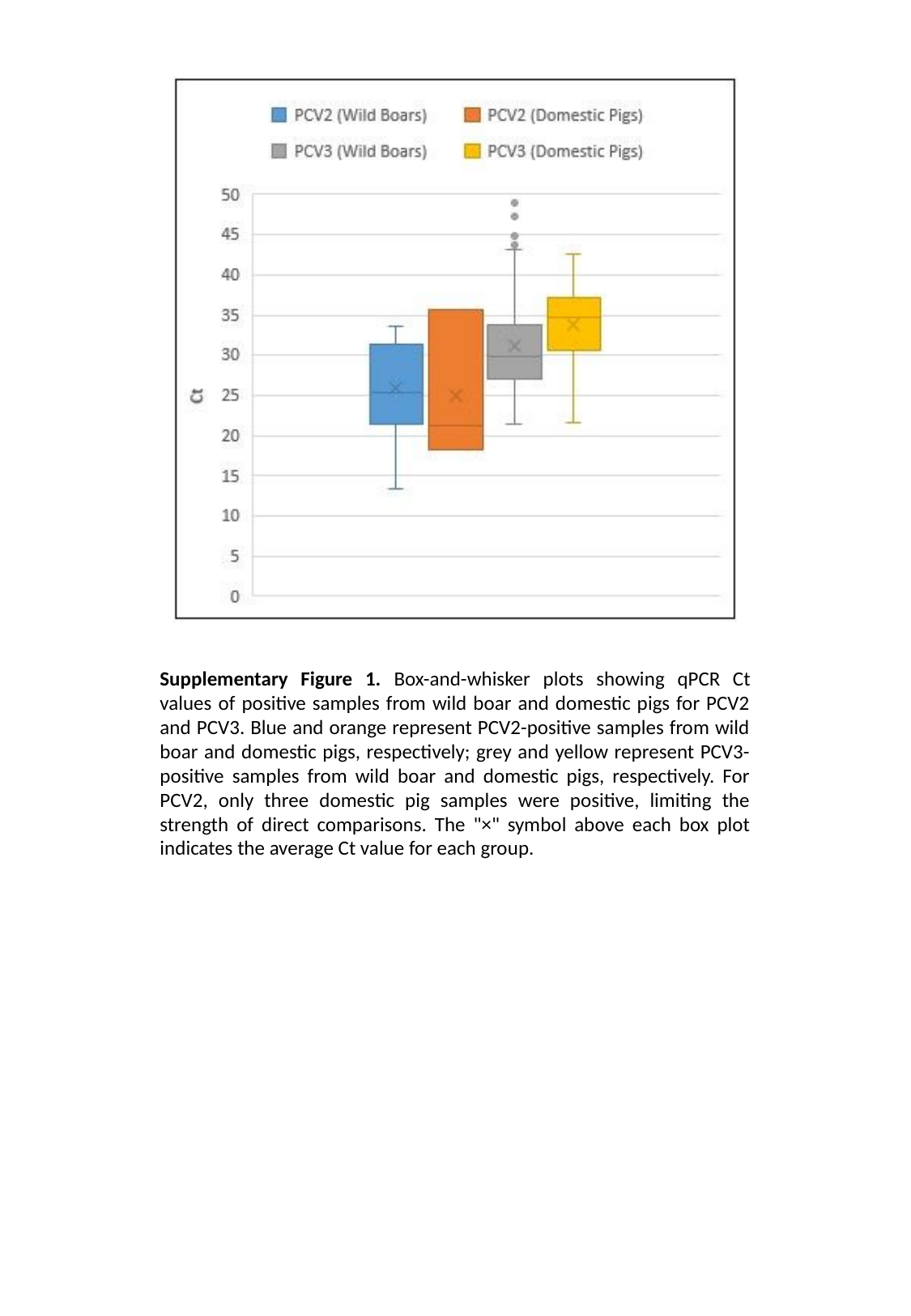

Supplementary Figure 1. Box-and-whisker plots showing qPCR Ct values of positive samples from wild boar and domestic pigs for PCV2 and PCV3. Blue and orange represent PCV2-positive samples from wild boar and domestic pigs, respectively; grey and yellow represent PCV3-positive samples from wild boar and domestic pigs, respectively. For PCV2, only three domestic pig samples were positive, limiting the strength of direct comparisons. The "×" symbol above each box plot indicates the average Ct value for each group.
